# The relationship between uric acid and bone mineral density in the intermediate stage of CKD 1–3

**DOI:** 10.1186/s12882-024-03650-7

**Published:** 2024-07-09

**Authors:** Ruiling Yang, Ning Ding, Jiao Qin, Hongchun Peng

**Affiliations:** 1 Department of Nephrology, The Affiliated Changsha Central Hospital, Hengyang Medical School, University of South China, 161 Shaoshan South Road, Changsha, Hunan 410004 China; 2 Department of Emergency Medicine, The Affiliated Changsha Central Hospital, Hengyang Medical School, University of South China, 161 Shaoshan South Road, Changsha, Hunan 410004 China; 3 Department of Orthopedics, The Affiliated Changsha Central Hospital, Hengyang Medical School, University of South China, 161 Shaoshan South Road, Changsha, Hunan 410004 China

**Keywords:** Uric acid, Bone mineral density, Chronic kidney disease, Gender

## Abstract

**Background:**

Some studies have suggested that uric acid has antioxidant properties that can prevent bone loss, but the relationship between uric acid and bone mineral density is controversial. The aim of this study was to investigate the relationship between UA and BMD in patients with CKD stage 1–3.

**Methods:**

We extracted 13,047 participants from the NHANES database, including 7342 male subjects and 5705 female subjects. Weighted multiple linear regression analysis was used to investigate the correlation between UA and BMD in patients with CKD stages 1–3.

**Results:**

In patients with CKD stage 1–3, UA was significantly correlated with BMD. In the male group, UA was positively associated with BMD (β, 7.94 [95%CI, 4.95, 10.94]). In the female group, there was a negative relationship between them (β, -5.33 [95%CI, -8.77, -1.89]). The relationship between UA and BMD in male group showed an inverted U-shaped curve, and UA was positively correlated before 6.1 mg/dl and negatively correlated after 6.1 mg/dl. The relationship was basically negative in the female group.

**Conclusions:**

For the patients with CKD stage 1–3, the relationship between UA and BMD showed an inverted U-shaped curve in the males, while the relationship was largely negative in the females.

**Supplementary Information:**

The online version contains supplementary material available at 10.1186/s12882-024-03650-7.

## Background

The end product of purine breakdown in the human body is uric acid (UA) [1]. UA is eliminated primarily by the kidneys and intestines [2]. The glomeruli filter out most UA, then the ultimate excretion of UA in the urine is determined by the degree of reabsorption and secretion of the renal tubules. The S1 segment of proximal renal tubule is responsible for UA reabsorption. In S2, UA reabsorption is less than its secretion [3]. A large number of initial studies have shown that high blood UA is a related risk factor for a variety of diseases, including metabolic syndrome and cardiovascular disease [1]. Over the past few decades, the incidence of hyperuricemia has increased rapidly worldwide. A number of observational articles have shown that elevated UA was independently associated with hypertension, gout, metabolic syndrome, urolithiasis and type 2 diabetes mellitus (T2DM), which may lead to an increased risk of adverse health conditions, including disability and death [4].

However, recently some studies have shown that UA can protect cells against oxidative damage of powerful endogenous antioxidants [1]. Higher serum UA levels are associated with the slow progression of certain diseases, such as Parkinson's disease and Huntington's disease [5]. Several studies have shown that UA may act as an antioxidant by scavenging free radicals, affecting life expectancy in patients with various diseases such as diabetes and obesity [6–8]

According to the World Health Organization (WHO), osteoporotic fracture is a public health priority due to its high morbidity and mortality, and the increase of fracture has correlation with lower bone mineral density (BMD) [9]. Increased survival with improved chronic kidney disease (CKD) treatment has made CKD-Mineral and Bone Disorder (CKD-MBD) a relevant "non-traditional" risk factor for morbidity and mortality in this population. CKD-MBD was characterized by disturbances of calcium, phosphorus, parathyroid hormone (PTH) and vitamin D, leading to physiological bone turnover, mineralization, longitudinal growth and volume disturbances. Histological findings are described as osteodystrophy and extraosseous mineralization in soft tissue and vascular structures [10]. Cross-sectional studies have shown a strong and independent association between serum UA levels and BMD. With UA levels increasing, the incidences of osteoporosis or fracture decrease significantly [10]. However, another study came to a different conclusion: there is no significant correlation between uric acid and bone density in adult Americans [11]**.**

To further explore the relationship between uric acid and bone mineral density, in this study, we intends to investigate the relationship between UA and BMD in patients with CKD stage 1–3 based on a large public database.

## Methods

### Study design and participants

**Fig. 1:**
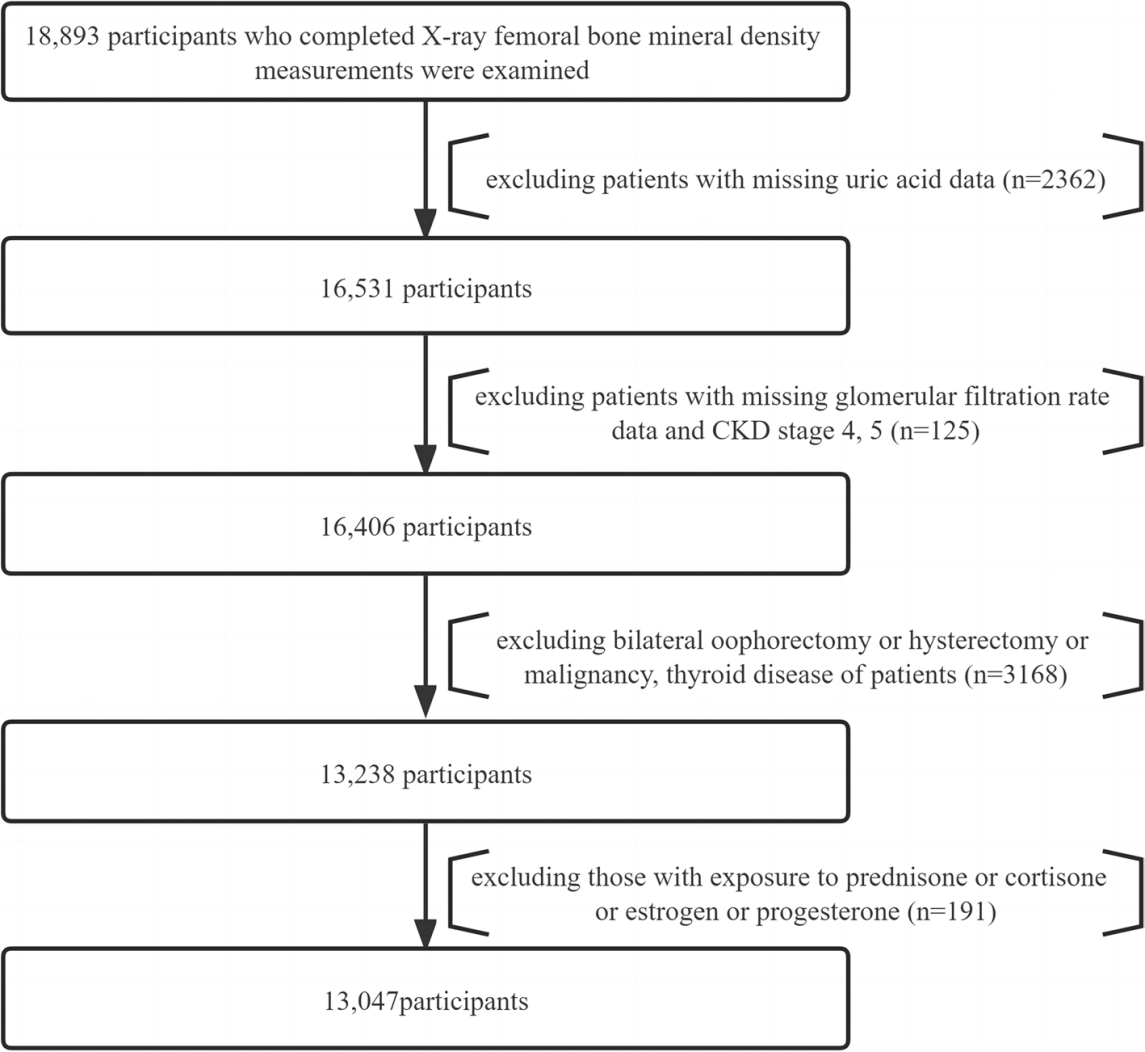
Flowchart of the study design and participants excluded from the study

NHANES was a survey of the United States population nutritional and health status of the research project [12]. It was calculated on a two-year period. Each cycle had a certain number of people and collected indicators related to their sociology, eating habits, disease conditions, lifestyle, and blood tests. In our study, data were collected for analysis from four cycles: 2007–2008, 2009–2010, 2013–2014, and 2017–2018 (the lack of data from 2011–2012 and 2015–2016 was due to the absence of femoral bone density examination in these two cycles). To investigate the relationship between UA and BMD in patients with CKD stages 1–3, we designed a cross-sectional study. 18,893 participants who completed X-ray femoral BMD measurements were examined. We excluded patients without UA data (*n* = 2362); patients without glomerular filtration rate (GFR) data and CKD stage 4, 5 (*n* = 125); patients with bilateral oophorectomy, patients with hysterectomy, patients with malignant tumors, patients with thyroid disease (*n* = 3168); patients exposed to hormone or progesteron or estrogene therapy (*n* = 191). Eventually, 13,047 patients with CKD stages 1–3 participated in the final analysis (Fig. 1).

### BMD and CKD

In this study, BMD refers to the total femur BMD, and its data was obtained by measuring the femur by dual energy X-ray absorptiometry (DXA). Femoral bone density scans were performed using three Hologic QDR-4500A fan-beam densitometers by trained and certified radiologists. Instrument by the manufacturer requires a longitudinal monitoring scanning femoral prosthesis every week. CKD is a kind of secondary to renal function and/or significant structural change of clinical syndrome, characterized by irreversibility and slow progression [13]. CKD is determined when an adult patient meet one of two criteria: 1) GFR less than 60ml/min/1.73m^2^; 2) The GFR was no less than 60ml/min/1.73m^2^, but there was evidence of renal structural damage [13], GFR is calculated according to MDRD equation: GFR = 186 × SC^−1.154^ × Age^−0.203^ × (0.742 if female) [14].

### Sociodemographic and lifestyle factors

Demographic variation values in this study included: sex, age, and race. Lifestyle factors including smoking, drinking and total physical activity (TPA). Smoking status includes current smokers, former smokers, non-smokers and unrecorded persons. Alcohol use is classified as current, abstinent or unrecorded. Data from TPA include the following types of physical activity: transportation, occupational and recreational. The sum of the three physical activities is TPA [15, 16]. Detailed definitions can be found in the supplementary materials.

### Metabolic, clinical laboratory indicators, and dietary factors

In our study, metabolic factors and clinical laboratory markers included hypertension, diabetes, total cholesterol, body mass index (BMI), serum vitamin D2 + D3, glomerular filtration rate (GFR), diuretic treatment, uric acid-lowering therapy and UA. Participants will be determined to have diabetes if they meet one of four criteria: 1) fasting blood glucose ≥ 7.0 mmol/L, 2) glycosylated hemoglobin ≥ 6.5% [17]. 3) 2-h Oral Glucose Tolerance Test blood glucose ≥ 200mg/dL, 4) taking hypoglycemic agent and insulin. Participants defined as having high blood pressure met one of the following 3 criteria: 1) taking antihypertensive medication; 2) diagnosed with hypertension; 3) Systolic or diastolic blood pressure ≥ 140mmHg or 90mmHg for 3 consecutive times [18]. Patients were defined as being on diuretic therapy if they were taking furosemide or hydrochlorothiazide diuretics. Patients were defined as undergoing uric acid-lowering therapy if they were taking allopurinol or febuxostat. Dietary variables included total daily protein and total calcium intake. BMI, total calcium intake and total protein definitions are detailed in the supplementary material.

### Statistical analysis

Use appropriate weights, stratification and clustering in data analysis. Since NHANES performs physical exams and lab analysis at the Mobile Exam Center (MEC). Therefore, we weighted the data using the “Full sample 2 year MEC exam weight” weights. We used a weighted mean (95% confidence interval (CI)) for continuous variables and a weighted percentage (95% CI) for categorical variables. In this study, multiple linear regression model was used to analyze the association between UA and BMD in stage 1–3 CKD patients, and stratified analysis was conducted by gender. In order to discover the nonlinear association between UA and BMD, smooth curve fitting was performed. All variables were included in the model in order to adjust potential confounding factors and by gender stratification analysis. Use of statistical package R (http://www.R-project.org) and empowerments statistics (http://www.empowerstats.com) for data analysis. When *p* < 0.05, the difference was considered statistically significant.

## Results

### Subjects characteristics

Characteristic of the research subjects are summarized in Table 1. A total of 13,047 subjects (7,342 males and 5,705 females) participated in the final analysis. Non-Hispanic whites were 65.89% (66.59% in males and 65.00% in females); the average age of the study population was 45.23 years for males and 43.94 years for females. The prevalence of hypertension were 35.00% in males and 29.85% in females, the prevalence of diabetes were 13.62% in males and 9.47% in females. In the male group, the smoking and drinking rates were 45.83% and 54.43%, respectively. In the female group, the rate of smoking and drinking was 33.27% and 40.71%, respectively. Compared with females, males had significantly higher prevalence of diabetes, hypertension, lower serum levels of vitamin D2 + D3, total cholesterol, higher levels of protein intake, calcium intake, BMI, BMD and UA (*P* < 0.05). The BMD of males and females were: 1027.61mg/cm^2^, 920.09mg/cm^2^; UA were: 6.03mg/dL, 4.68mg/dL; Creatinine were: 84.83umol/L, 66.11umol/L. The majority of participants did not receive diuretic therapy or uric acid-lowering therapy. (Table 1). We have drawed the scatter plot to further show the relationship between UA and BMD, as shown in the figure below, the image shows that the data is clustered, and the relationship between UA and BMD is not completely linear ( Supplementary Fig. 1).

**Table 1:** Description of participants based on gender

	Male (*n* = 7342)	Female (*n* = 5705)	*P*-value
Age(years)	45.23 (44.59,45.87)	43.94 (43.32,44.56)	< 0.0001
Race			0.0005
Mexican American	9.84 (7.77,12.38)	9.09 (7.06,11.62)	
Other Hispanic	5.68 (4.37,7.36)	6.17 (4.86,7.82)	
Non-Hispanic White	66.59 (62.53,70.42)	65.00 (60.86,68.92)	
Non-Hispanic Black	10.05 (8.75,11.53)	11.99 (10.26,13.97)	
Other Race	7.84 (6.56,9.33)	7.75 (6.58,9.11)	
Smoking status			< 0.0001
Current-smoking	20.51 (19.05,22.04)	16.19 (14.67,17.84)	
Former smoking	25.32 (23.82,26.87)	17.08 (15.78,18.47)	
Non-smoking	43.75 (41.49,46.03)	54.93 (52.63,57.20)	
Not recorded	10.43 (9.75,11.15)	11.80 (10.69,13.01)	
Alcohol consumption			< 0.0001
No drinking	26.39 (24.65,28.20)	30.36 (28.52,32.27)	
Drinking	54.43 (52.37,56.48)	40.71 (38.68,42.78)	
Not recorded	19.18 (18.09,20.32)	28.93 (27.13,30.79)	
Total physical activity			< 0.0001
Inactive participants	26.38 (24.91,27.92)	38.85 (37.21,40.52)	
Active participants	73.62 (72.08,75.09)	61.15 (59.48,62.79)	
Diabetes			< 0.0001
No	86.38 (85.29,87.40)	90.53 (89.46,91.51)	
Yes	13.62 (12.60,14.71)	9.47 (8.49,10.54)	
Hypertension			< 0.0001
No	64.92 (63.17,66.64)	70.04 (68.48,71.55)	
Yes	35.00 (33.29,36.76)	29.85 (28.35,31.39)	
Not recorded	0.07 (0.03,0.17)	0.11 (0.05,0.26)	
Total Cholesterol( mmol/L)	4.90 (4.87,4.93)	5.03 (4.99,5.06)	< 0.0001
BMI (kg/m^2^)	27.92 (27.72,28.13)	27.38 (27.14,27.63)	0.0006
Serum vitamin D2 + D3	67.47 (66.06,68.88)	70.63 (69.05,72.21)	0.0001
Protein intake(g/day)	96.09 (94.68,97.50)	68.80 (67.76,69.85)	< 0.0001
Calcium intake(mg/day)	1079.36 (1057.79,1100.93)	873.98 (852.83,895.13)	< 0.0001
BMD(mg/cm^2^)	1027.61 (1022.25,1032.96)	920.09 (915.27,924.91)	< 0.0001
Uric acid (mg/dL)	6.03 (5.99,6.07)	4.68 (4.64,4.73)	< 0.0001
GFR (ml/min/1.73m^2^)			0.3491
≥ 90	45.15 (43.42,46.90)	44.10 (41.84,46.37)	
< 90	54.85 (53.10,56.58)	55.90 (53.63,58.16)	
Creatinine(umol/L)	84.83 (84.32,85.35)	66.11 (65.44,66.77)	< 0.0001
Diuretic treatment			0.4820
Yes	7.67 (6.93,8.48)	8.04 (7.11,9.08)	
No	92.33 (91.52,93.07)	91.96 (90.92,92.89)	
Uric acid-lowering therapy			< 0.0001
Yes	1.87 (1.44,2.42)	0.13 (0.07,0.26)	
No	98.13 (97.58,98.56)	99.87 (99.74,99.93)	

For continuous variables: survey-weighted mean (95% CI), *P*-value was by survey-weighted linear regression

For categorical variables: survey-weighted percentage (95% CI), *P*-value was by survey-weighted Chi-square test

*BMD* Bone mineral density, *BMI* Body mass index, *GFR* Glomerular filtration rate

### The relationship between UA and BMD in patients with CKD stages 1–3

A weighted multiple linear regression analysis was performed on UA and BMD of CKD stages 1–3 patients in different models, and the results were shown in Table 2. In Model I, unadjusted, there was a significant positive correlation between UA and BMD (β, 30.54 [95% CI, 28.17, 32.91]); In Model II, sociodemographic and lifestyle factors were adjusted, including gender, age, race, smoking, alcohol consumption, total physical activity. It could be found that UA and BMD still showed a significant positive correlation (β, 18.22 [95%CI, 15.68, 20.77]). Model III further adjusted for metabolic, clinical laboratory measures, and dietary factors (BMI, total cholesterol, diabetes mellitus, hypertension, serum vitamin D2 + D3, protein intake, calcium intake, GFR, diuretic treatment, uric acid-lowering therapy) based on model II, and there were still positive correlation (β, 2.96 [95%CI, 0.55, 5.36]). In order to reflect the reliability of our research conclusions, we also used femoral neck bone density for analysis, and the results were basically consistent (Supplementary Table 1).

**Table 2:** Result of multiple linear regression analysis between UA and BMD in CKD 1–3 stage

Exposure	Model I(β,95%CI,P)	Model II(β,95%CI,P)	Model III(β,95%CI,P)
UA(mg/dL)	30.54 (28.17, 32.91) < 0.0001	18.22 (15.68, 20.77) < 0.0001	2.96 (0.55, 5.36) 0.0214

For BMD: survey-weighted β (95%CI) *p*-value

Model I was adjusted for: none

Model II was adjusted for: gender, age, race, smoking, alcohol consumption, total physical activity in addition to model I

Model III was adjusted for: body mass index, total cholesterol, diabetes, hypertension, serum vitamin D2 + D3, protein intake, calcium intake, GFR, diuretic treatment, uric acid-lowering therapy in addition model II

*CI* Confidence interval, *UA* Uric acid, *BMD* Bone mineral density, *CKD* Chronic kidney disease, *GFR* Glomerular filtration rate

### Gender differences in the relationship between UA and BMD in patients with CKD stages 1–3

Multiple linear regression analysis of UA and BMD in CKD stages 1–3 stratified by sex are shown in Table 3. After adjusting for race, age, alcohol consumption, smoking, BMI, total physical activity, total cholesterol, hypertension, diabetes, serum vitamin D2 + D3, calcium intake**,** protein intake, GFR, diuretic treatment, uric acid-lowering therapy, UA and BMD were found to be significantly higher in the male group than in the female group, and UA was positively correlated with BMD in male group (β, 7.94 [95%CI, 4.95, 10.94]). In female group, UA was negatively correlated with BMD(β, -5.33 [95%CI, -8.77, -1.89]).

**Table 3:** Result of multiple linear regression analysis between UA and BMD in CKD 1–3 stage stratified by gender

Exposure	Male (*n *= 7342)	Female (*n* = 5705)
UA(mg/dL)	7.94 (4.95, 10.94) < 0.0001	-5.33 (-8.77, -1.89) 0.0046

For BMD: survey-weighted β (95%CI) *p*-value

Adjust for: age, race, smoking, alcohol consumption, total physical activity, body mass index, total cholesterol, diabetes, hypertension, serum vitamin D2 + D3, protein intake, calcium intake, GFR, diuretic treatment, uric acid-lowering therapy

*CI* confidence interval, *UA* Uric acid, *BMD* Bone mineral density, *CKD* Chronic kidney disease, *GFR* Glomerular filtration rate

**Fig. 2:**
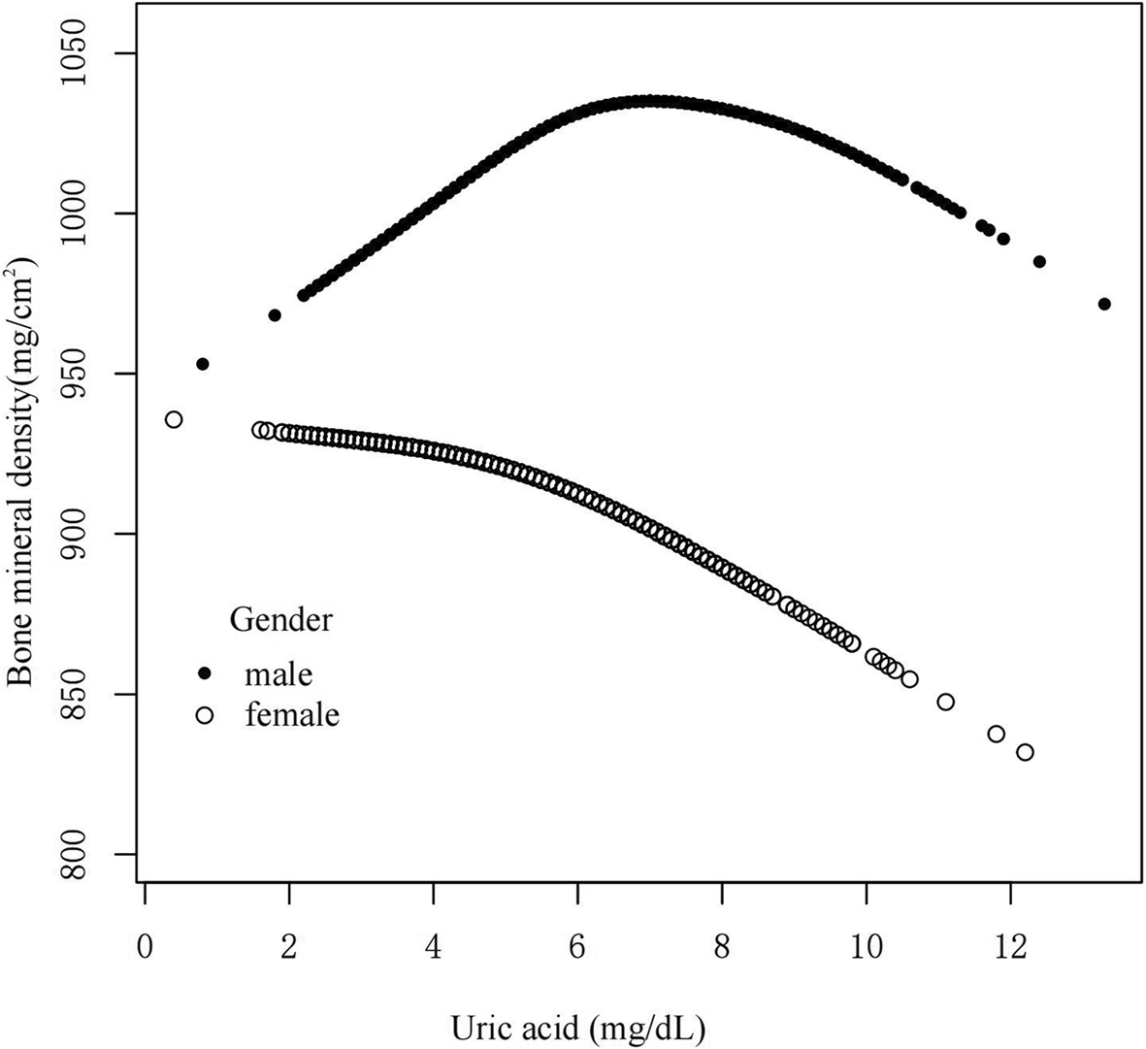
A smooth curve fitting for the relationship between UA and BMD in CKD 1–3 stage stratified by gender. adjust for: age, race, smoking, alcohol consumption, total physical activity, body mass index, total cholesterol, diabetes, hypertension, serum vitamin D2 + D3, protein intake, calcium intake, GFR, diuretic treatment, uric acid-lowering therapy. Abbreviations: UA, uric acid; BMD, Bone mineral density; CKD, chronic kidney disease; GFR, glomerular filtration rate

**Fig. 3:**
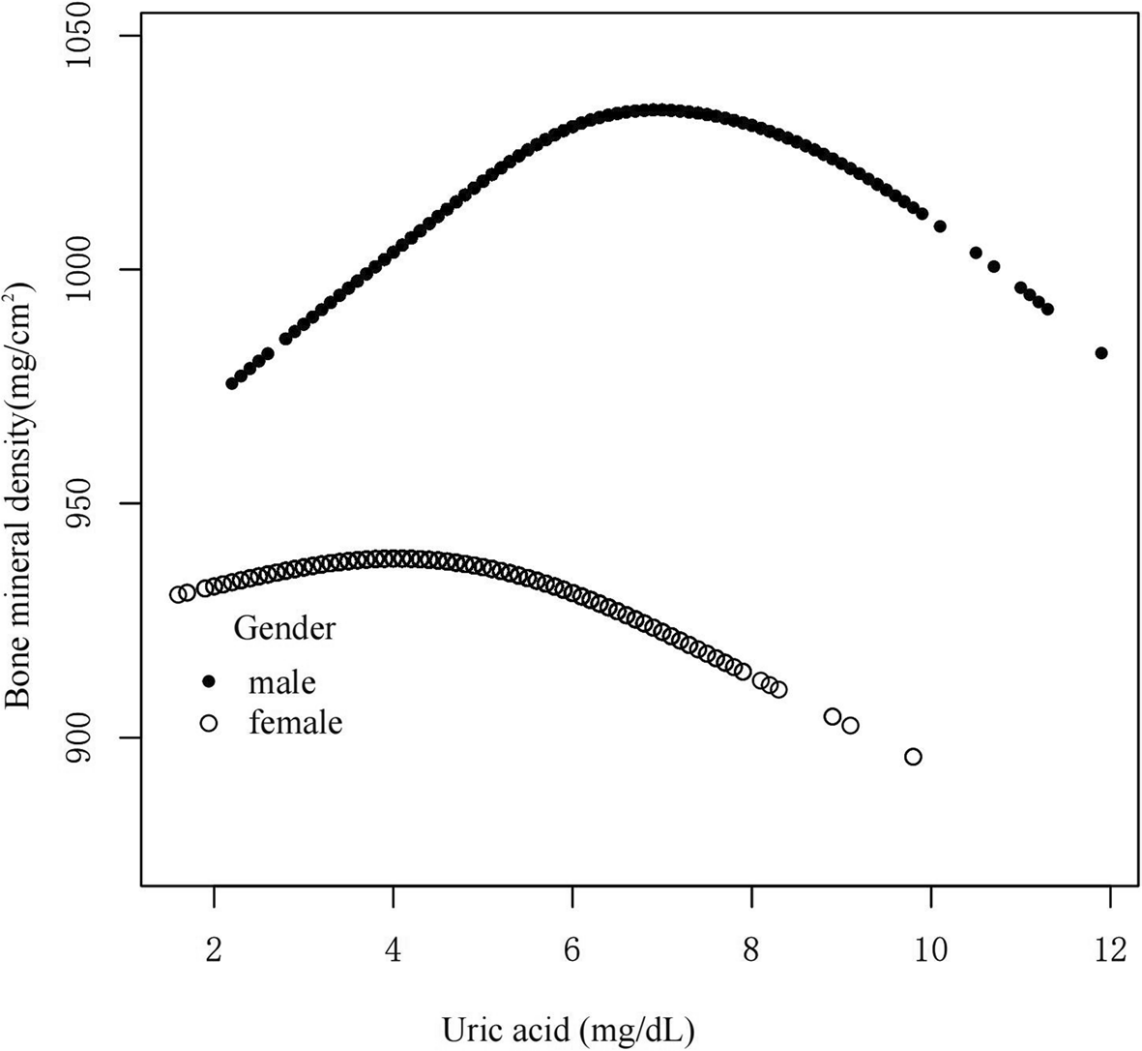
A smooth curve fitting for the relationship between UA and BMD in CKD 1 stage stratified by gender. adjust for: age, race, smoking, alcohol consumption, total physical activity, body mass index, total cholesterol, diabetes, hypertension, serum vitamin D2 + D3, protein intake, calcium intake, GFR, diuretic treatment, uric acid-lowering therapy. Abbreviations: UA, uric acid; BMD, Bone mineral density; CKD, chronic kidney disease; GFR, glomerular filtration rate

**Fig. 4:**
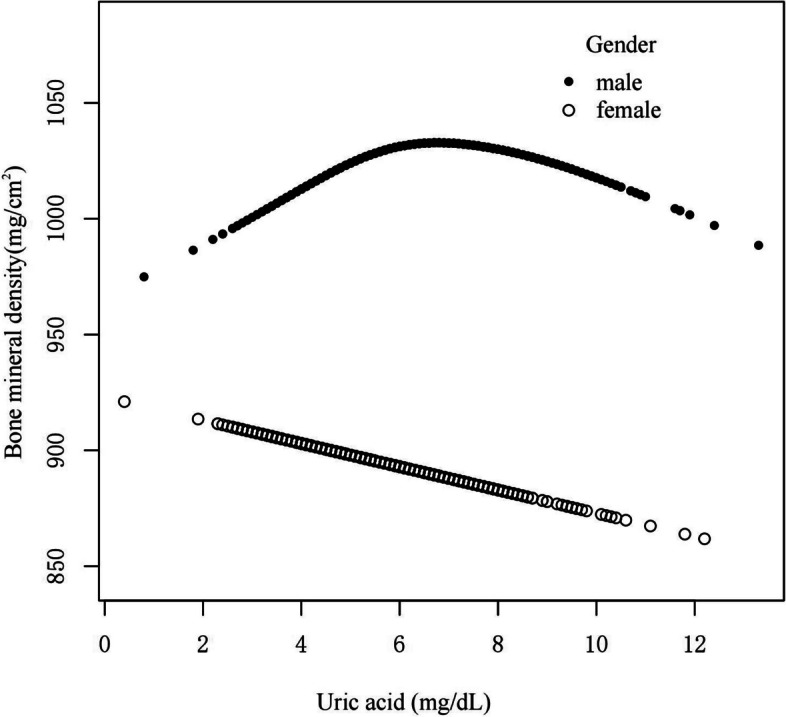
A smooth curve fitting for the relationship between UA and BMD in CKD 2–3 stage stratified by gender. adjust for: age, race, smoking, alcohol consumption, total physical activity, body mass index, total cholesterol, diabetes, hypertension, serum vitamin D2 + D3, protein intake, calcium intake, GFR, diuretic treatment, uric acid-lowering therapy. Abbreviations: UA, uric acid; BMD, Bone mineral density; CKD, chronic kidney disease; GFR, glomerular filtration rate

To discover non-linear relationships, smooth curve fitting was performed for male and female groups. The male group (Fig. 2) showed an inverted U-shaped curve. The correlation in the female group was essentially linear. We also used femoral neck bone mineral density as the dependent variable to explore their relationship, and their relationship was basically consistent with the trend when total femur BMD was used as the dependent variable (Supplementary Fig. 2). We further used piecewise linear regression to analyze the threshold effect of UA on BMD in males with CKD stages 1–3 (Table 4). In males, when UA ≤ 6.1 mg/dL, UA and BMD was significantly positive correlation(β, 16.14 [95%CI, 11.48, 20.80]). When UA > 6.1 mg/dL, UA was negatively correlated with BMD (β, -6.58 [95%CI, -10.92, -2.25]). Smooth curve fitting was performed for both male and female patients with CKD1 stage (Fig. 3), with an inverted U-shaped curve in the male group and a slightly inverted U-shaped curve in the female group. Smooth curve fitting was performed on the male and female groups of patients with CKD stages 2–3 (Fig. 4), and the male group still showed an inverted U-shaped curve. The correlation was linear in the female group.

**Table 4:** Threshold effect analysis of UA on BMD in CKD 1–3 stage using piecewise linear regression in male

Inflection point of UA	β (95% CI)	*P*-value
≤ 6.1 mg/dL	16.14 (11.48, 20.80)	< 0.0001
> 6.1 mg/dL	-6.58 (-10.92, -2.25)	0.0029

For BMD: survey-weighted β (95%CI) *p*-value

Adjust for: age, race, smoking, alcohol consumption, total physical activity, body mass index, total cholesterol, diabetes, hypertension, serum vitamin D2 + D3, protein intake, calcium intake, GFR, diuretic treatment, uric acid-lowering therapy

*UA* Uric acid, *BMD* Bone mineral density, *CKD* chronic kidney disease, *GFR* Glomerular filtration rate

## Discussion

In this survey study, the association between UA and BMD in a population with CKD stages 1–3 in large crowds were investigated (*n* = 13,047). After adjusting for possible confounding factors, a positive correlation between BMD and UA was obtained. In the analysis after further gender stratification, the results showed that in the male group, the relationship between UA and BMD was positively correlated in the range of UA ≤ 6.1 mg/dL, and negatively correlated beyond this range. In the female group, the relationship between the two was essentially negative.

This result may be explained by the following potential mechanisms. Extracellular UA has antioxidant properties and can effectively remove free radicals from human plasma, antioxidants prevent bone loss [19]. Studies have shown that during the degradation of UA, intracellular oxygen free radicals are generated, The increase of fibroblast growth factor-23 (FGF-23), decrease of calcitriol, increase of parathyroid hormone, increase of phosphate, associated bone disease, and vascular calcification are the main biochemical characteristics of mineral metabolism changes [20], the up-regulation of FGF-23 is one of the pathogenesis mechanisms of CKD-MBD [21, 22]. In a similar study of patients with CKD, FGF-23 was associated with UA levels independently, and the association was stronger in males than in females [23]. Another study found that in healthy people, there was a positive correlation between both [24]. Considering that UA may influence BMD by influencing the above related metabolic factors in CKD population. Elevated PTH in patients with CKD is associated with abnormal vitamin d levels. High parathyroid hormone causes calcium to fall off the bone, thus weakening the bone and participating in the development of renal bone disease [25]. Hyperphosphatemia is common in patients with CKD, and the increase of calcium and phosphorus products promotes extraosseous mineralization [26]. Although both PTH and phosphate are involved in the development of CKD-MBD, due to the lack of this part of data in the database, we excluded patients with stage 4–5 CKD to reduce the impact of this study. In addition, other studies have suggested an association between hyperuricemia and high bone mineral density, possibly due to the inhibition of osteoclast bone resorption due to the potential antioxidant effect of uric acid [27]. At the same time, there are other studies that show that higher UA levels are associated with lower testosterone levels, and testosterone deficiency is also a cause of osteoporosis [28]. Researches show that gender differences can be observed in bone structure, osteoporosis pathophysiology and other aspects [29]. Although epidemiological studies have shown that hyperuricemia increases steadily with age in all populations [30], some studies have shown different rates of hyperuricemia in older males and postmenopausal females, suggesting that gonadal hormones may play a role. Females estrogen prevents osteoporosis, and postmenopausal females constitute an estrogen deficient population [31]. It is susceptible to oxidative stress of many molecules, which is the main cause of high risk of osteoporosis [32]. Although we came to a different conclusion than the studies mentioned earlier [11], we believe this is due to differences in the population samples collected, adjusted variables, and data used in the two studies. And the relationship between UA and BMD needs to be further investigated in specific populations in the future. Our study has the advantages of large sample size and novelty: this was the first study to investigate the relationship between UA and BMD in CKD1-3 population and to explore its sex difference. But there are still some limitations. First, it was a cross-sectional study. Therefore, the causal relationship between UA and BMD cannot be confirmed. Second, because the database did not include relevant data, the postmenopausal women were not further grouped. Third, there were residual confounders that were not included in the model for adjustment, such as parathyroid hormone, rheumatoid arthritis, and hip osteoarthritis. Finally, some of the variables in this study were based on patient self-reports, so the data may be affected by subjective recall bias.

## Conclusions

For the patients with CKD stage 1–3, the relationship between UA and BMD showed an inverted U-shaped curve in the males, while the relationship was largely negative in the females.

### Supplementary Information

Supplementary Material 1.

Supplementary Material 2. The scatter plot concerning BMD and UA values. The scatter plot concerning BMD and UA values.

Supplementary Material 3. A smooth curve fitting for the relationship between UA and femoral neck BMD in CKD 1-3 stage stratified by gender

Supplementary Material 4.

## Data Availability

The datasets used and/or analyzed during the present study were availed by the corresponding author on reasonable request.
