# Supplementary material for: The relationship between uric acid and bone mineral density in the intermediate stage of CKD 1–3
Source: BMC Nephrol. 2024 Jul 9;25:219. doi: 10.1186/s12882-024-03650-7 (PMC11234712; doi:10.1186/s12882-024-03650-7)
Supplement: Supplementary file 1 — Supplementary Material 1. [file 12882_2024_3650_MOESM1_ESM.docx]

**Detailed rules for the definition of each variable**

**The definition of Lifestyle factors**

Lifestyle factors included smoking, alcohol consumption and total physical activity (TPA). Smoking status was classified according to the smoking questionnaire: current smoker, former smoker, non-smoker and unrecorded. Non-smoking is defined as smoking fewer than 100 cigarettes in a lifetime. Alcohol consumption was classified as drinking, abstaining and unrecorded according to the alcohol questionnaire. Of these, drinking alcohol at least 12 times a year was defined as alcohol consumption. The TPA data was built on the Global Physical Activity Questionnaire, which includes three types of physical activity: recreational, occupational and transportation, divided into vigorous and moderate categories according to the intensity of each physical activity. The sum of the three physical activities is TPA(multiplied by 2 if the physical activity is vigorous)^[1,2]^. According to the 2018 Physical Activity Guidelines for Americans, participants with TPA ≥150 minutes are defined as active participants^[3]^.

**The definition of metabolic, clinical laboratory indicators and dietary factors**

Metabolic factors and clinical laboratory markers included diabetes, hypertension, body mass index (BMI), total cholesterol, glomerular filtration rate (GFR), serum vitamins D2+D3, and UA. Participants who meet one of the following 4 criteria will be defined as having diabetes: 1) fasting blood sugar ≥ 7.0 mmol/L, 2) glycohemoglobin ≥ 6.5%, 3) 2-hour Oral Glucose Tolerance Test blood sugar ≥ 200mg/dL, 4) taking hypoglycemic agent and insulin. Participants who meet one of the following 3 criteria will be defined as having hypertension: 1) taking antihypertensive drugs; 2) diagnosed with hypertension; 3) 3 consecutive systolic blood pressure ≥140 mmHg or diastolic blood pressure ≥90 mmHg^[4]^. BMI is calculated by dividing weight (in kilograms) by the square of height (in meters). Dietary variables included total daily protein and total calcium intake. Dietary investigators conducted two 24-hour dietary recall records. Total protein and total calcium intakes were the average of protein and calcium intakes recorded from two dietary recalls. GFR was calculated according to MDRD equation: GFR=186 × SC^−1.154^ × Age^−0.203^× (0.742 if female)^[5]^. **Patients were defined as being on diuretic therapy if they were taking furosemide or hydrochlorothiazide diuretics. Patients were defined as undergoing uric acid-lowering therapy if they were taking allopurinol or febuxostat.**

**References**

[1] Divney AA, Murillo R, Rodriguez F, Mirzayi CA, Tsui EK, Echeverria SE. Diabetes Prevalence by Leisure-, Transportation-, and Occupation-Based Physical Activity Among Racially/Ethnically Diverse U.S. Adults. Diabetes Care. 2019. 42(7): 1241-1247.

[2] Kim D, Konyn P, Cholankeril G, Ahmed A. Physical Activity Is Associated With Nonalcoholic Fatty Liver Disease and Significant Fibrosis Measured by FibroScan. Clin Gastroenterol Hepatol. 2022. 20(6): e1438-e1455.

[3] Piercy KL, Troiano RP, Ballard RM, et al. The Physical Activity Guidelines for Americans. JAMA. 2018. 320(19): 2020-2028.

[4] Yoon SS, Carroll MD, Fryar CD. Hypertension Prevalence and Control Among Adults: United States, 2011-2014. NCHS Data Brief. 2015. (220): 1-8.

[5] Levey AS, Coresh J, Greene T, et al. Expressing the Modification of Diet in Renal Disease Study equation for estimating glomerular filtration rate with standardized serum creatinine values. Clin Chem. 2007. 53(4): 766-72.
