# Supplementary figures and images for: The relationship between uric acid and bone mineral density in the intermediate stage of CKD 1–3
Source: BMC Nephrol. 2024 Jul 9;25:219. doi: 10.1186/s12882-024-03650-7 (PMC11234712; doi:10.1186/s12882-024-03650-7)

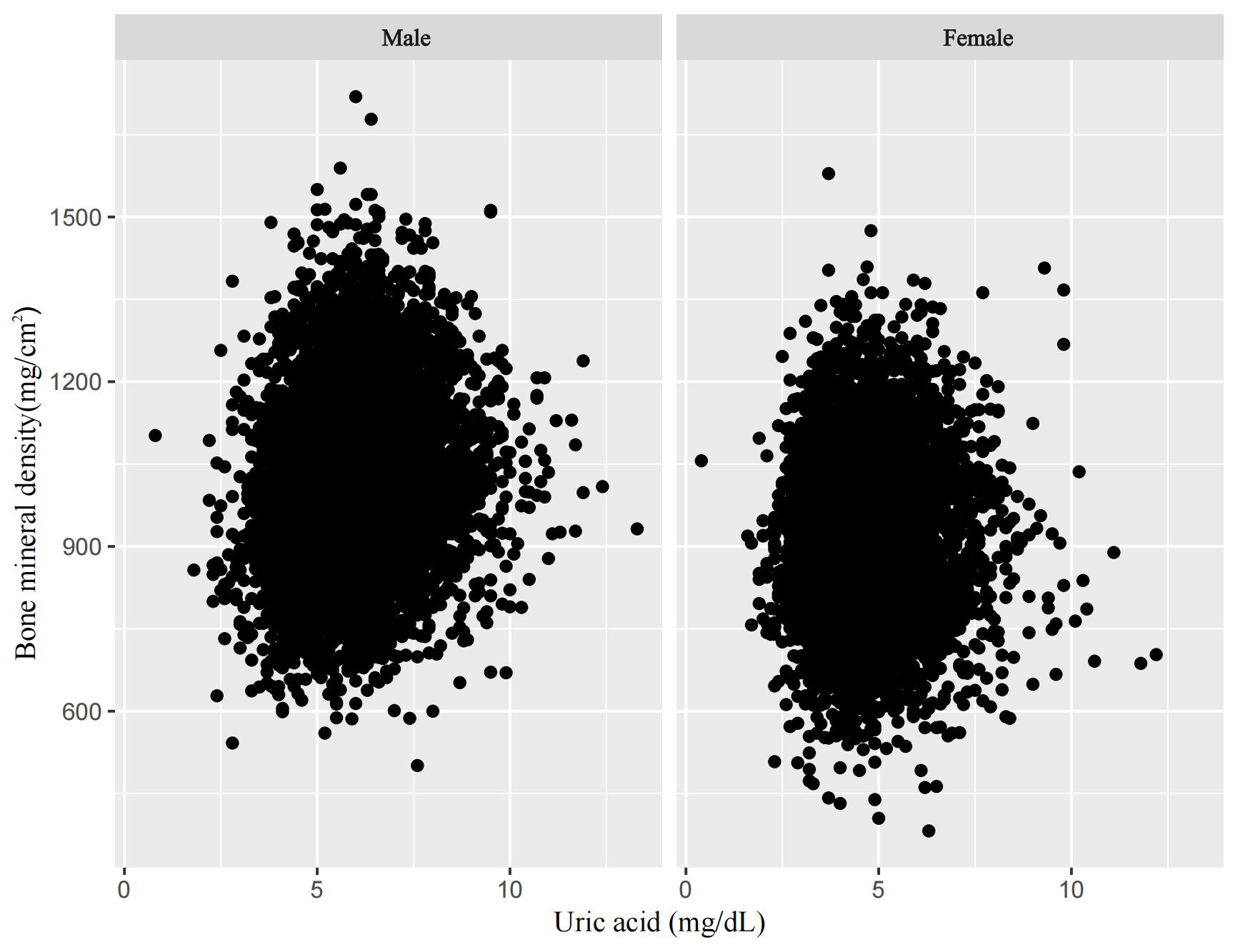

Supplement: Supplementary file 2 — Supplementary Material 2. The scatter plot concerning BMD and UA values. The scatter plot concerning BMD and UA values. [file 12882_2024_3650_MOESM2_ESM.jpg]

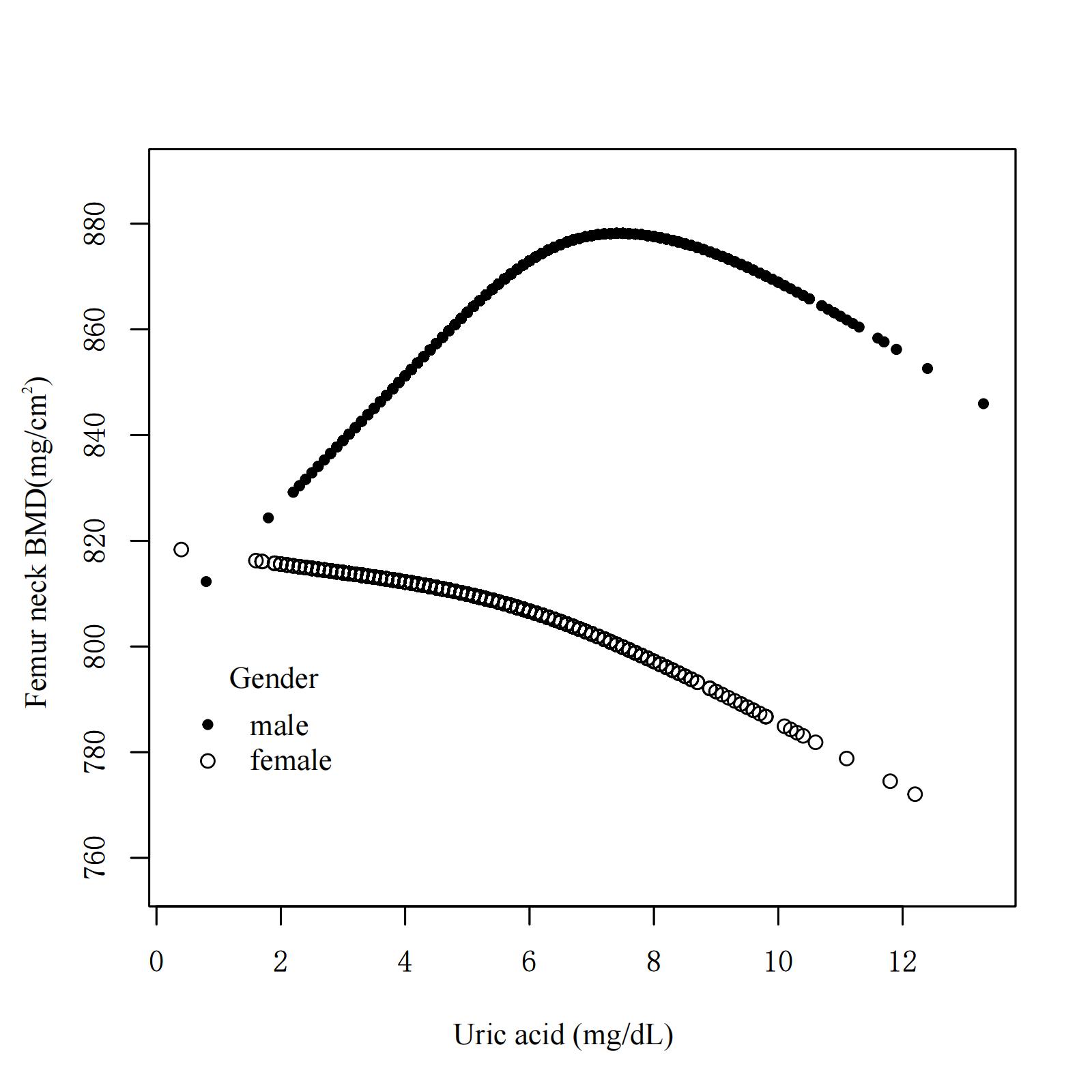

Supplement: Supplementary file 3 — Supplementary Material 3. A smooth curve fitting for the relationship between UA and femoral neck BMD in CKD 1-3 stage stratified by gender [file 12882_2024_3650_MOESM3_ESM.jpg]
