# Supplementary material for: The relationship between uric acid and bone mineral density in the intermediate stage of CKD 1–3
Source: BMC Nephrol. 2024 Jul 9;25:219. doi: 10.1186/s12882-024-03650-7 (PMC11234712; doi:10.1186/s12882-024-03650-7)
Supplement: Supplementary file 4 — Supplementary Material 4. [file 12882_2024_3650_MOESM4_ESM.docx]

**Supplementary Table 1.** Result of multiple linear regression analysis between UA and **femoral neck** BMD in CKD 1-3 stage.

| Exposure | Model I(β,95%CI,P) | Model II(β,95%CI,P) | Model III(β,95%CI,P) |
| --- | --- | --- | --- |
| UA(mg/dL) | 18.73 (16.44, 21.02) <0.0001 | 16.70 (14.15, 19.24) <0.0001 | 4.37 (1.77, 6.97) 0.0023 |

Abbreviations: CI, confidence interval; UA, uric acid; BMD, Bone mineral density; CKD, chronic kidney disease; GFR, glomerular filtration rate.

For **femoral neck** BMD : survey-weighted β (95%CI) p-value.

Model I was adjusted for: none.

Model II was adjusted for: gender, age, race, smoking, alcohol consumption, total physical activity in addition to model I.

Model III was adjusted for: body mass index, total cholesterol, diabetes, hypertension, serum vitamin D2 + D3, protein intake, calcium intake, GFR, diuretic treatment, uric acid-lowering therapy in addition model II.
